# Nanostructured Bioaerogels as a Potential Solution for Particulate Matter Pollution

**DOI:** 10.3390/gels9070575

**Published:** 2023-07-14

**Authors:** Wafa Mustafa Saleh, Mardiana Idayu Ahmad, Esam Bashir Yahya, Abdul Khalil H.P.S.

**Affiliations:** 1 Environmental Technology Division, School of Industrial Technology, Universiti Sains Malaysia, Penang 11800, Malaysia; 2 Renewable Biomass Transformation Cluster, School of Industrial Technology, Universiti Sains Malaysia, Penang 11800, Malaysia; 3 Bioprocess Technology Division, School of Industrial Technology, Universiti Sains Malaysia, Penang 11800, Malaysia; 4 Green Biopolymer, Coatings and Packaging Cluster, School of Industrial Technology, Universiti Sains Malaysia, Penang 11800, Malaysia; akhalilhps@gmail.com; 5 Bioresource Technology Division, School of Industrial Technology, Universiti Sains Malaysia, Penang 11800, Malaysia

**Keywords:** particulate matter removal, filtration, biopolymers, bioaerogels, nanostructured

## Abstract

Particulate matter (PM) pollution is a significant environmental and public health issue globally. Exposure to high levels of PM, especially fine particles, can have severe health consequences. These particles can come from a variety of sources, including natural events like dust storms and wildfires, as well as human activities such as industrial processes and transportation. Although an extensive development in air filtration techniques has been made in the past few years, fine particulate matter still poses a serios and dangerous threat to human health and to our environment. Conventional air filters are fabricated from non-biodegradable and non-ecofriendly materials which can cause further environmental pollution as a result of their excessive use. Nanostructured biopolymer aerogels have shown great promise in the field of particulate matter removal. Their unique properties, renewable nature, and potential for customization make them attractive materials for air pollution control. In the present review, we discuss the meaning, properties, and advantages of nanostructured aerogels and their potential in particulate matter removal. Particulate matter pollution, types and sources of particulate matter, health effect, environmental effect, and the challenges facing scientists in particulate matter removal are also discussed in the present review. Finally, we present the most recent advances in using nanostructured bioaerogels in the removal of different types of particulate matter and discuss the challenges that we face in these applications.

## 1. Introduction

Air filtration biomaterials have recently become a research hotspot on account of the increasing attention paid to the global air quality problem [1]. Particulate matter (PM) is the pollution made up of particles (tiny pieces) of solids that are in the air that may include: dust, dirt, etc. [2]. PM air pollutants result from both natural and anthropogenic sources. Increased concentration of PM in the surrounding atmospheric environment devastates human health [3]. Particulate matter has been divided into three different groups: the first group is PM10, which includes coarse particles and relatively large particles. PM10 mostly describes inhalable particles, including those particles less than 10 micrometers in diameters [4]. The second group is fine particle matter (PM2.5), which includes tiny particles that can cause haziness to the air upon their elevation. PM2.5 are able to travel deeply into the respiratory tract, reaching the lungs [5]. Exposure to fine particles can cause short-term health effects such as eye, nose, throat, and lung irritation, coughing, sneezing, runny nose, and shortness of breath [6]. The third group is ultrafine particles (PM0.1), which have an aerodynamic diameter of around 0.1 μm [7]. All three groups of PM form a real danger to the human health since they have the ability to penetrate the human body through the respiratory system. Therefore, the removal, or at least the reduction, of PM has become a necessity for a safe environment.

**Figure 1:**
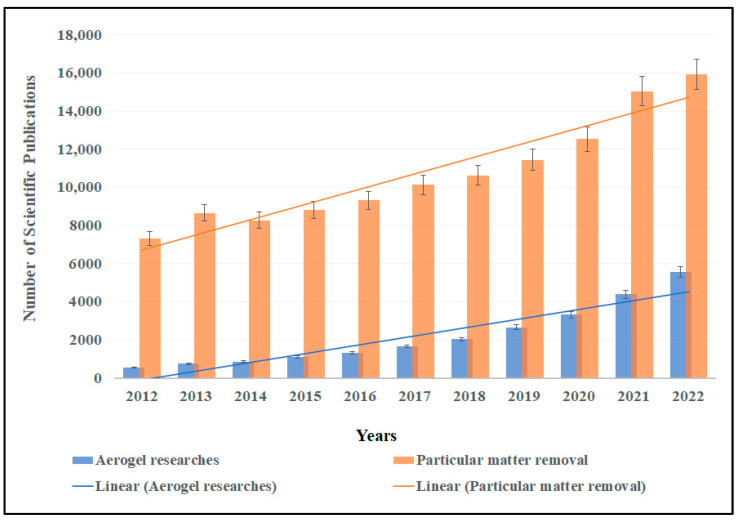
Number of scientific studies in the last ten years about aerogel and particular matter removal. Search conducted through Science Direct database on 17 April 2023.

Air filters have been used to capture different types of PM, including PM10 and PM2.5 [8,9]. The PM filter industry has had tremendous progression, even though some concerns and challenges still exist. First of all, the technology that has been used for existing PM filters is lacking ecofriendly characteristics since remarkable amounts of solvents and toxic materials are used for the electrospinning process that negatively influence human health and the surrounding environment [10]. Moreover, the accumulating discarded filters with high volumes of trapped PM constitute a direct threat to the environment. Besides the toxicity, those PM filters were designed to capture PM at a small range of concentration which is 1000 mg within the size of a cubic centimeter. In this case, those filters are facing a great challenge to capture PM within highly polluted environments. The capture of particulate matter via conventional and common purification technologies basically focuses on size-dependent mechanisms, which in most cases is fairly difficult due to the extremely small particle size of particulate matter and its ultra-low mass [11]. Conventional filters have been made of several materials such as fabrics, wool, cotton, etc., mostly without considering their pore size or removal efficiency. In the research of Liu et al. [12], filters made of a polyamide-56 nanofiber/net had a high filtration effectiveness of 99.995% for most of air pollutants. Although the filter was able to remove almost all the particulate matter, the issue of using toxic and non-ecofriendly material is still present. Other scientists have used PLA/PMMA composite nanofibers to solve the fine particle matter issue and claim that they removed 99.5% of PM2.5 using synthetic stimulations [13]. Liu et al. [14] developed a superhydrophobic filter using a mix of synthetic polymers and they were able to remove up to 96% of the PM2.5. Bioaerogels have been proposed for PM filtration as ecofriendly and sustainable functional materials [15]. Bioaerogels are known to have various advantages over synthetic-based materials such as regeneration, biocompatibility, biodegradability, low density, high porosity, and a large specific surface area [16,17]. They are three-dimensional nanoporous structures with high surface area and porosity, derived from biopolymers such as chitosan, cellulose, alginate, or proteins [18,19]. These aerogels exhibit unique properties that make them attractive for various applications across multiple industries. Thus, extensive studies have been conducted regarding the fabrication, modification, and application of aerogels in the past few years, which can be observed from the increased number of scientific publications in the past ten years together with concerns about the adverse health effects of particulate matter pollution (Figure 1).

In the early periods of the air industrial revolution, filters were used to purify the atmosphere from particulate matter [20]. Air filters were developed rapidly during that period of time using different precursor materials. However, currently, there is growing interest in finding inexpensive, abundant, and effective materials to use as effective air filtration with much focus on natural organic polymers, mainly from agriculture. Several researchers have prepared effective biopolymer-based aerogel filters for particulate matter removal [21,22]. In this review, we discuss the meaning, properties, and advantages of nanostructured aerogels and their potential in particulate matter removal. Particulate matter pollution, types and sources of particulate matter, health effects, environmental effects and the challenges facing scientists in particulate matter removal are also discussed in the present review. Finally, we present the most recent advances in using nanostructured bioaerogels in the removal of different types of particulate matter and discuss the challenges that we face in these applications. The review will conclude by summarizing the potential of biopolymer aerogels as a sustainable and effective solution for PM removal. It will emphasize the advantages of biopolymer aerogels over other materials, highlighting their potential for further research and development in the field of air pollution control. By critically evaluating the performance of biopolymer aerogels in comparison to other works, this review paper aims to provide a comprehensive understanding of their potential for PM removal. The insights gained from this review will contribute to the development of innovative and efficient materials in addressing the pressing issue of particulate matter pollution.

## 2. Nanostructured Bioaerogels

Nanostructured aerogels are a type of nanomaterial with porose diameters ranging from 1 to 100 nm [23]. They are considered excellent alternative adsorbents and filters due to their extremely large surface area, exceptional chemical surface properties, and broad range of selectivity for pollutants [24]. The pore size of nanostructured aerogels can be modified by changing the precursor material(s) or the fabrication technique or through the addition of reinforcement materials. Nanostructured aerogels have been modified using various materials including polymers, inorganic carbon, metal oxides, and silica [25]. Aerogels have been extensively synthesized from natural biomass to have ecofriendly properties, but the hydrophilic nature of biomass and most biopolymers is still a great challenge. Most prepared biomass aerogels including white protein, whey protein, starch, Arabic gum, chitosan, alginate, and pectin lack hydrophobicity, which make aerogels lose their architecture in very humid conditions [26,27]. Several researchers aimed to improve the stability and mechanical properties of biopolymer aerogels by using different chemical modifications, but this raises the cost of production and produces non-ecofriendly materials.

### 2.1. Preparation of Nanostructured Bioaerogels

**Figure 2:**
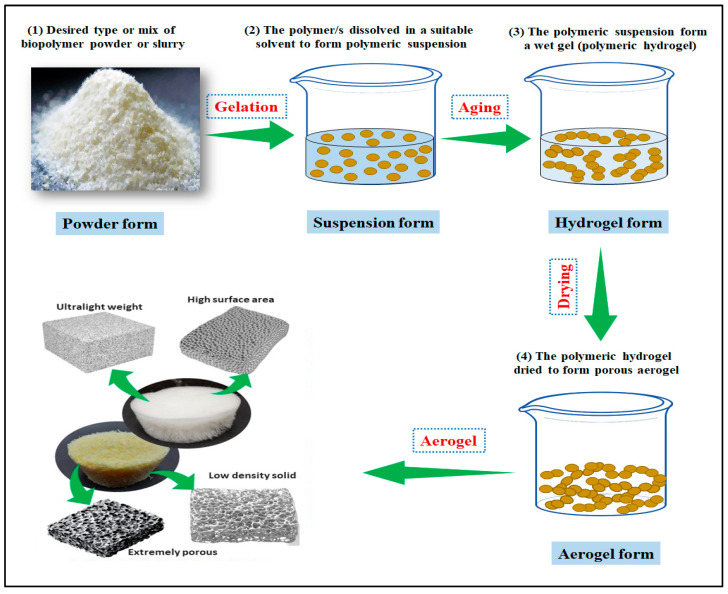
Illustration of the basic principle of nanostructured bioaerogel fabrication.

In recent years, there have been notable developments in the formation of various forms of aerogels, such as biomass-derived, inorganic carbon-based, polymer-based, and silica-based aerogels, among others [25]. Aerogels can be made from a wide range of materials, and the properties of the aerogel depend on the material used [28]. However, the absence of unique characteristics in a single material restricts the versatility of many pure aerogels. As a result, composite aerogels offer a solution for numerous potential applications by allowing for the enhancement, introduction, and development of new materials for a variety of new uses. The past few years witnessed the development of several techniques for the fabrication of biopolymer-based nanostructured bioaerogels. But all of these techniques follow the same basic principle: the gelation of polymeric suspension, aging, and finally drying (Figure 2) [29].

Aerogels with different properties can be obtained by varying the precursor material(s) and/or the parameters of these three steps [28,30]. The unique properties of nanostructured bioaerogels arise from the extraordinary flexibility as well as the resilience of the sol–gel process to form the polymeric wet gel, followed by the drying stage. Drying the wet gel (hydrogel) is a critical process that affects the properties of the material. Different drying methods have been reported to result in different forms of materials; supercritical and freeze drying mostly result in the formation of hydrogel [31,32], while ambient drying produces xerogel [33]. In the gelation phase, the dissolution of the biopolymer(s) in the solvent occurs, which then leads to network formation (crosslinking) in the second phase in the aging process, which is critical to form a homogenous nanostructured aerogel [34]. Some biopolymers are able to directly form networks in the gelation phase such as chitosan and gelatin, while other biopolymers like cellulose require the addition of curing factors or crosslinker(s) to form the network [35,36]. Finally, removing the solvent is known as the drying phase, which is referred to as the gel–aerogel transition [37]. The fabrication techniques of nanostructured aerogels can be divided into two categories; the first category is conventional techniques which include freeze drying [16], gas foaming [38], phase separation [39], and electrospinning [40]. These techniques are extensively discussed by Abdul Khalil et al. [41]. Recent years have witnessed the development of the second category, faster and computer-aided techniques able to professionally design the properties of nanostructured aerogels. These techniques are known as rapid prototyping techniques and include 3D printing [42], fused deposition modeling [43], selective laser sintering [44], and stereolithography [45]. These techniques offer facile fabrication without the need for any complex tools or equipment where the biopolymers are used as ink (bio-injected ink). The properties of nanostructured aerogels such as porosity, shape, pore size, and volume as well as mechanical properties can be all adjusted by varying the ratio of the precursor materials and changing the preparation conditions [46].

### 2.2. Properties of Nanostructured Bioaerogels

Nanostructured bioaerogels are a special type of porous materials that possess unique properties depending on the precursor material(s). These aerogels are typically made from polysaccharides like cellulose, starch, chitosan, alginate, and pectin, which are abundant and renewable sources of materials that can replace petroleum-based products [47]. Due to their abundance, biodegradability, regeneration, and sustainability, bioaerogels are gaining popularity and are being developed as a replacement for traditional aerogels [27]. These aerogels have also been reported to possess remarkable air-purifying properties [48]. However, the mechanical properties of bioaerogels require improvement, and there is potential for the further exploration of their ability to adsorb PM2.5 [49]. Although natural materials are low cost, abundant, ecofriendly, and support the proper utilization of waste, their high water and moisture absorption is inferior compared to the efficiency of synthetic materials [49,50]. Their main disadvantages stem from their poor oleophilic/hydrophobic properties. In order to improve these characteristics, combined technologies, including the sol–gel technique and plasma treatment for achieving hydrophobic biopolymeric aerogels, are hypothesized to be stable in water and to have higher capacity for PM removal, especially in humid condition [51].

The specific properties of the aerogels depend on the type of biopolymer used and the preparation method employed. Nanostructured cellulose aerogel has several unique properties; it has an extremely low density, which makes it one of the lightest solid materials available [52]. Its density typically ranges from 0.01 to 0.5 g/cm^3^, which is much lower than most other materials [53]. Owing to its nanopores, nanostructured cellulose aerogel has an extremely high surface area per unit volume. Nanostructured cellulose aerogel has excellent thermal insulation properties, making it useful as a building insulation material or as a protective coating for industrial equipment [54]. Despite its low density, nanostructured cellulose aerogel has been reported to have high mechanical strength and can withstand significant compression without breaking, in addition to high water absorption due to its porous structure [55]. This property makes it useful in various applications such as water treatment, where it can absorb contaminants from water. Nanostructured chitosan aerogel has been reported to have high porosity, typically in the range of 80–99% depending on the concentration and preparation approach. Takeshita et al. [56] reported that the porosity of aerogel is determined by its preparation method and it can be controlled by adjusting various factors such as the concentration of chitosan, the type and concentration of the crosslinking agent, the solvent used, and the drying method. Despite its high porosity, nanostructured chitosan aerogel has good mechanical strength and can withstand compression without breaking [57]. Chitosan aerogel was also reported to have good thermal insulation properties due to its low thermal conductivity in addition to moderate antimicrobial properties, which makes nanostructured chitosan aerogel useful for biomedical applications [58]. Nanostructured alginate aerogel also has a similar porosity to cellulose and chitosan in addition to good mechanical strength, and it can withstand compression without breaking. Alginate can undergo ionotropic gelation, which means that it can form a gel when exposed to divalent cations such as calcium [59]. Overall, these properties make nanostructured biopolymer aerogels a promising material for particulate matter removal and air filtration applications.

### 2.3. Applications of Nanostructured Bioaerogel

Nanostructured bioaerogels have been experimented, used, and proposed for several applications that require the special performance of functional materials. They have been extensively used in many medical applications including drug delivery, tissue scaffolding, biosensing, and wound-healing applications [60]. Nanostructured bioaerogels have also been utilized in several environmental applications, especially in water treatment, for the removal of different pollutants including organic dyes, heavy metals, toxic substances, pesticides, herbicides, etc. [23]. Air purification has been also benefited from the development of these functional materials. The ability to be modified and the unique properties of their surface functional groups have promoted nanostructured bioaerogels in different applications [23]. Refer to Table 1 for the illustration of using nanostructured bioaerogels in different applications.

## 3. Particulate Matter Pollution

Particulate matter is one of the hazardous pollutants is that inhaled by humans and causes series health issues. Particulate matter, also known as particle pollution, refers to tiny particles of solid or liquid matter that are suspended in the air we breathe [76]. These particles can be of different sizes, shapes, and chemical compositions, and can come from natural sources like dust and wildfires, as well as human activities like burning fossil fuels and industrial processes [77]. To reduce the risks associated with particulate matter, efforts are underway to control and regulate emissions from industrial and transportation sources, as well as to improve air quality monitoring and warning systems. In the early periods of the air industrial revolution, filters were used to purify the atmosphere from PM [20]. Air filters were developed rapidly during that period of time to avoid or eliminate the adverse health effects of different types of particulate matter as described in the following sections.

### 3.1. Types and Sources of Particulate Matter

**Figure 3:**
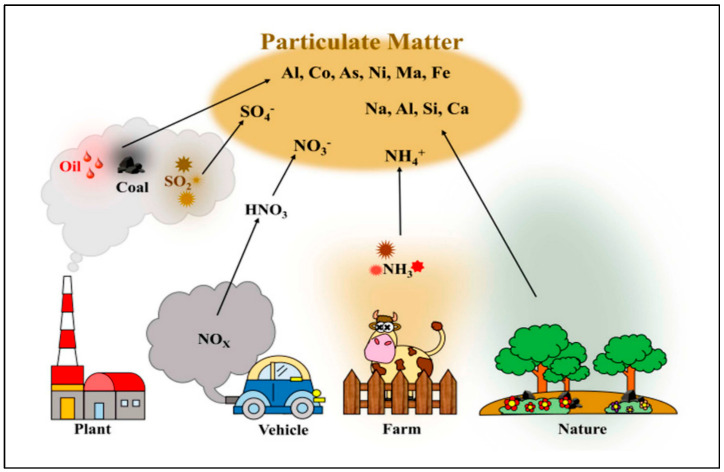
Schematic drawing of different sources of particulate matter in our environment. Adapted with permission from [92].

Particulate matter has been classified by aerodynamic diameter into three different groups: PM10 (≤10 microns), PM2.5 (≤2.5 microns), and PM1.0 (≤1.0 microns) [78]. All three types of PM form a real danger to human health since they have the ability to penetrate the human body through the respiratory system [79]. The sources of particulate matter significantly vary across locations for several reasons including the precursor material, emission sources, dispersion patterns, and distinct climatic conditions [5,80]. However, the source can be either natural or anthropogenic. It has been reported that PM2.5 levels are highly affected by biomass burning, vehicle traffic, ship emissions, power plants, dust resuspension, industrial emissions, and aircraft emissions [81]. It is well known that this fine particulate matter is basically composed of several undetermined fractions with different shapes and sizes. It is mostly formed from fuel emissions of vehicles in addition to the wear and tear of many auto parts [82]. It has been reported that the main components of particulate matter are polycyclic aromatic hydrocarbons [83], black carbon [84], volatile organic hydrocarbons [85], aryl hydrocarbons [86], organic compounds [87], inorganic ions [88], minerals [89], and biological materials [90]. These components are responsible of more than 85% of the total mass of particulate matter in the air [91]. Incomplete fossil fuel combustion was also reported to generate particulate matter in addition to biomass burning, vehicle emissions, and industrial emissions, as presented in Figure 3 [92].

The composition of particulate matter can vary significantly depending on its source, its location, and the time of the year [93]. Organic compounds including carbonaceous materials derived from incomplete combustion processes, such as fossil fuel combustion, biomass burning, and cooking, are the most common type of PM, which can be primary (emitted directly into the atmosphere) or secondary (formed through chemical reactions in the atmosphere) [93]. Elemental carbon is another type of solid carbonaceous component of PM that is primarily emitted from the combustion of fossil fuels, biomass burning, and industrial processes [94]. Particulate matter can also contain trace amounts of metals such as lead (Pb), arsenic (As), cadmium (Cd), nickel (Ni), and others. These metals can originate from industrial emissions, vehicle exhaust, combustion processes, and natural sources. Other chemical species such as sulfates, nitrates, ammonium, carbonates, and chlorides might also be present [77,95,96]. Other inorganic compounds are often associated with dust and soil particles that are resuspended into the air. PM can also contain biological particles such as pollen, spores, bacteria, and fungal spores. These particles are often associated with seasonal variations and can cause allergies and respiratory issues in susceptible individuals [97]. PM2.5 particles have been reported to have the ability to stay suspended in the air longer than the bigger types (PM10) [5]. The smaller the particular matter, the more toxic it is to humans due to its ability to penetrate into human bronchi and blood vessels. Therefore, the morbidity, toxicity, and mortality of fine particulate matter are significantly increased with long-term exposure to fine particulate matter [98].

### 3.2. Health Effects of Particulate Matter

**Figure 4:**
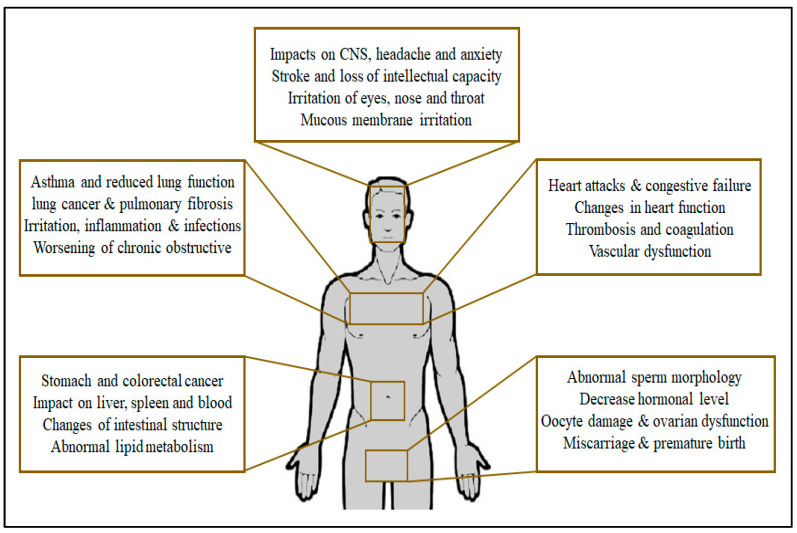
Illustration of the adverse health effects of particulate matter.

Particulate matter can pose a serious threat to human health, particularly if the particles are small enough to penetrate deep into the lungs and enter the bloodstream [99]. Exposure to high levels of particulate matter has been linked to a range of health problems, including respiratory and cardiovascular diseases, lung cancer, and premature death [100,101]. PM pollutants have been recently considered as a serious threat to public health due to their adverse health effects [102]. The inhalation of particulate matter can lead to respiratory diseases such as coughing, breathing difficulties, chronic bronchitis, and even cancer. The level of danger posed by these particles is inversely related to their size. Particles ranging from 5.5 to 9.2 μm in diameter can cause breathing difficulties by lodging in the nose and throat, while particles smaller than 5.5 μm can penetrate the breathing passages and cause more severe illnesses. The most perilous particles are those with a diameter of less than 1 μm, which can remain in air sacs and significantly increase the risk of lung cancer. PM has been reported to form a real danger to the human health since it has the ability to penetrate the human body through the respiratory system [103]. Exposure to fine particles can cause short-term health effects such as eye, nose, throat and lung irritation, coughing, sneezing, runny nose, and shortness of breath [2,104,105]. It was found that particulate matter particles are able to inhibit the biophysical functions of the lung surfactants by impeding molecular packaging in addition to the formation of surfactant–particle aggregates [106]. Fine particulate matter also can hinder and even prevent particle–cell interactions, which could modify the toxicological impact of the inhaled particles [107,108]. Thangavel et al. [5] extensively discussed the toxicity and adverse health effects of different types and sources of particulate matter. Figure 4 illustrates the adverse impacts of particulate matter on different parts of the human body.

## 4. Applications of Nanostructured Bioaerogel in Particulate Matter Removal

**Figure 5:**
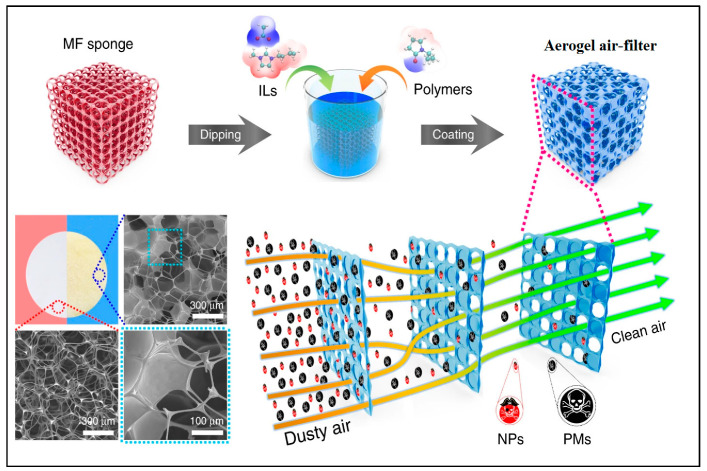
Illustration of multilayered aerogel-based air filtration approach. Melamine–formaldehyde (MF), ionic liquids (ILs), nanoscale particles (NPs), and particulate matter (PM). Adapted with permission from [102].

Nanostructured bioaerogel filtration was introduced as a new concept to purify the atmosphere that can overcome the limitations associated with conventional approaches. Nanostructured bioaerogels exhibit unique properties such as huge surface area, high porosity, and controlled pore size; biopolymer-based aerogels can be utilized in all types of PM air filtration [25,47]. Multilayered aerogel filters differ from conventional monolayered filters in that they can even eliminate ultrafine PM along with fine PM (Figure 5). For this reason, they were widely used in synthesizing aerogel-based filters [109,110].

### 4.1. Biomass-Based Nanostructured Aerogel

Biomass aerogel is a type of aerogel material that is derived from biomass, which refers to any organic material that is produced by living organisms or from their metabolic processes. Biomass can be derived from a wide range of sources, such as plant matter, agricultural waste, and industrial byproducts [111]. Biomass aerogels have several advantages over traditional aerogels, including lower cost, increased sustainability, and reduced environmental impact [112]. One of the key advantages of biomass aerogels is their potential to provide a sustainable alternative to traditional aerogel materials, which are often derived from non-renewable sources and can be expensive to produce. In a recent investigation, Wang et al. [113] prepared a novel environmentally friendly nanostructured aerogel by using konjac glucomannan as a precursor material and enhanced it with wheat straw. The bioaerogel was prepared by using the conventional approach of sol–gel followed by a freeze-drying process. The authors reported that the addition of wheat straw biomass into the aerogel enhanced its porosity from 50% to more than 88%. The filtration capacity of the aerogel also improved to 90.38%. Furthermore, the addition of wheat straw significantly enhanced the mechanical properties of the aerogel, which reported compressive strength, compression modulus, and elasticity of 501.56 Pa, 2000.66 Pa, and 0.603, respectively. By utilizing biomass, which is often a waste product or byproduct of other processes, biomass aerogels can be produced at a lower cost and with reduced environmental impact.

**Figure 6:**
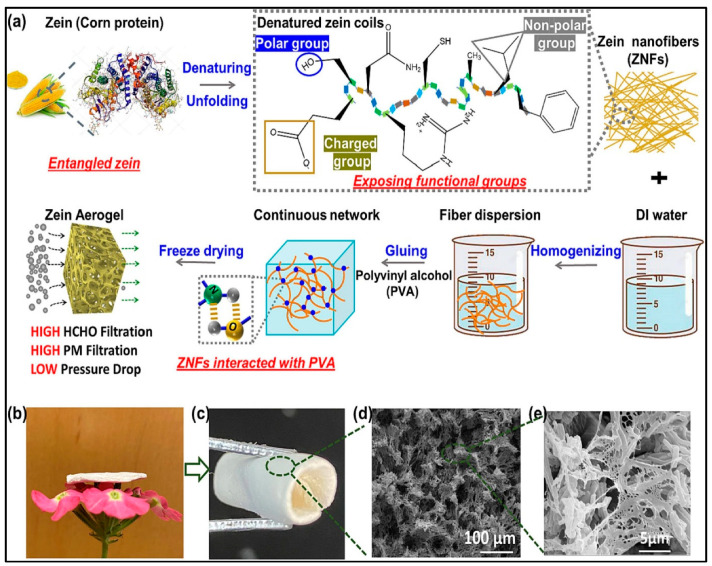
Preparation of corn protein nanostructured aerogels; (**a**) schematic drawing of the whole preparation process; (**b**) a diagram of the aerogel being placed on a flower to show the light weight; (**c**–**e**) the morphology and SEM images of the fabricated nanostructured aerogel. Adapted with permission from Lin et al. [116].

Overall, biomass aerogels are a promising new material with a wide range of potential applications and are an example of how sustainable biomaterials can be utilized in advanced materials science. Biomass nanostructured aerogel have shown great potential in particulate matter removal due to their high surface area, porous structure, and ability to adsorb pollutants. Wang et al. [114] fabricated another konjac glucomannan nanostructured aerogel for particulate matter removal using the conventional sol–gel and lyophilization methods. The authors reported that the addition of starch and gelatin into the aerogel could significantly enhance the filtration performance of the aerogel and increase its compressive strength. These polysaccharides increase the porosity of the aerogel, reduce the pore size, and thus enhance its filtration performance. The same authors also used wheat straw as a filler in the aerogel and reported that its addition decreased the filtration resistance and significantly enhanced the breathability of the aerogel. This could be attributed to the multi-cavities of wheat straw [115]. Wheat-straw-enriched bioaerogel exhibited 93.5% filtration efficiency of fine particulate matter (≥0.3 μm) and an air permeability 271.4 L/s·m^2^. Such nanostructured bioaerogel possessed a water contact angle of 105.4°, which shows its potential in resisting moisture and its workability even in humid conditions as an air filtration material. In a recent study, corn protein was used to fabricate nanostructured bioaerogels with controlled structures for particulate matter removal [116] (Figure 6). The authors enhanced the filtration properties of the aerogel by adding polyvinyl alcohol to glue dispersed corn protein nanofibers and form the bioaerogel. The aerogel exhibited high capturing properties for particulate matter; up to 99.52% of PM2.5 and 98.80% of PM0.3. The authors also stated that their nanostructured bioaerogel was able to eliminate formaldehyde by 87.41% at a low pressure drop. Overall, biomass aerogels have shown great potential in particulate matter removal due to their unique properties and versatility. Further research is needed to optimize their use for different applications and to address any potential limitations.

### 4.2. Cellulose-Based Nanostructured Aerogel

Cellulose is the most abundant biopolymer on earth. Cellulose aerogels have been extensively studied and used in several applications including particulate matter removal [117]. Cellulose is considered a great alternative for plastics and other fossil-oil-based materials that can alleviate environmental pollution [29]. Cellulose aerogels are made by first extracting cellulose from plants and then dissolving it in a solvent. The cellulose solution is then subjected to a process called gelation, where it is transformed into a gel-like substance. The gel is then dried under controlled conditions to remove the solvent, resulting in a highly porous, low-density aerogel material [118]. Cellulose aerogels are known for their unique properties, including high thermal insulation, high mechanical strength, and biodegradability. In the past few years, a huge number of functional cellulosed aerogels have been prepared, modified, and utilized in particulate matter removal. Xie et al. [65] recently fabricated a carbonized cellulose-based aerogel using cotton wastes as precursor materials. The authors claimed that their aerogel was able to significantly filtrate the particulate matter due to the grown molybdenum disulfide. Owing to the high specific surface area and the high electrical conductivity of the prepared bioaerogel, it forms a strong electrostatic force between the particulate matter particles and the aerogel, with more than 99.91% and 99.95% removal efficiency for PM2.5 and PM10, respectively [65].

**Figure 7:**
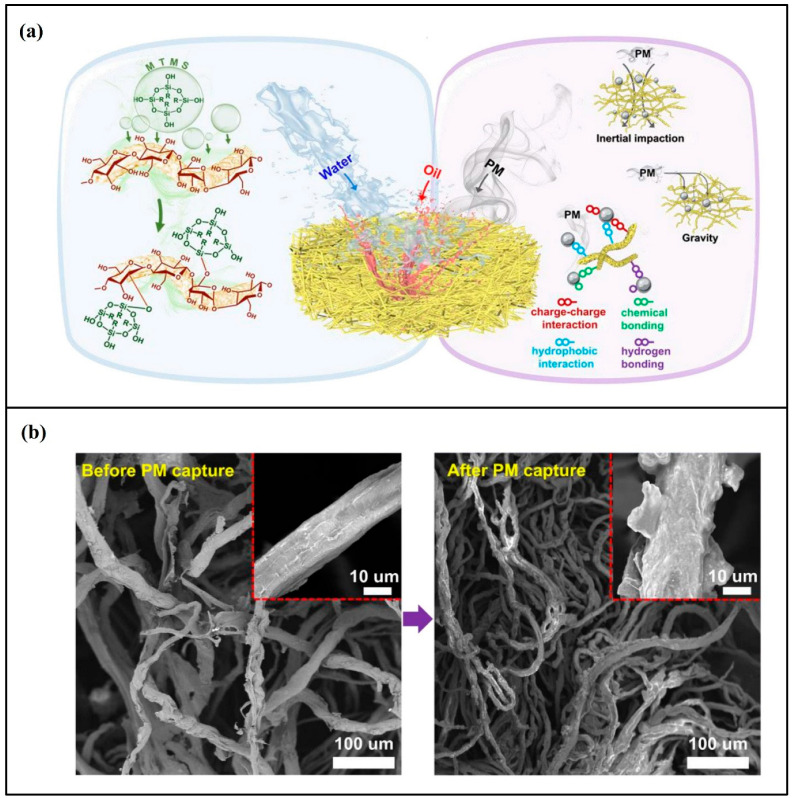
Hydrophobic nanocellulose aerogel for particulate matter removal; (**a**) Schematic drawing of the aerogel mechanism and (**b**) SEM images before and after PM capturing. Adapted from with permission Lyu et al. [119].

Bacterial cellulose-based aerogels were prepared using a directional ice-templated approach and tested for PM removal [22]. The authors modified bacterial cellulose to enhance the surface functional groups with reactive silane precursors. The aerogel exhibited excellent quantitative removal of PM (more than 95%). In a different study, Lyu et al. [119] used waste hemp oil in the fabrication of aerogel via dissolution in a precooled NaOH/urea system (Figure 7). The aerogel was highly hydrophobic and highly porose and exhibited elastic behavior. The authors reported that their aerogel had a high removal capacity at 94% for both PM2.5 and PM10. Nanocellulose aerogels exhibit smooth fibers rich in surface functional groups. After the filtration, these fibers were found to be fully covered by particulate matter particles at the end of the filtering process, which proves the excellent PM capturing ability of nanocellulose aerogels.

### 4.3. Chitosan-Based Nanostructured Aerogel

Chitosan is another polysaccharide obtained from shrimp, shellfish, and other crustacean shells [120]. It has been extensively used in different forms in air filtration due to its polarization ability, strong polarity, antimicrobial properties, biodegradability, and nontoxicity [121]. These unique properties make chitosan highly attractive in air filtration applications. Chitosan aerogels are highly porous, lightweight materials that have a large surface area and can be easily modified to have specific chemical and physical properties [122]. These properties make chitosan aerogels excellent candidates for air filtration applications. The air filtration process with chitosan-aerogel-based filters involves passing the contaminated air through the aerogel material, where particulate matters are trapped and removed. The highly porous nature of chitosan aerogels allows for a high airflow rate, resulting in efficient and effective air filtration [56]. Chitosan-aerogel-based air filters have several advantages over conventional air filters, including high filtration efficiency, low energy consumption, and a long service life. They also have potential applications in various fields, such as indoor air purification, industrial air filtration, and medical air filtration. Desai et al. [123] developed a chitosan-based nanofibrous filter through an electrospinning technique. The authors reported that the filtration efficiency of their fabrication was strongly associated with pore size and surface area. The authors claimed that their fabrication was able to eliminate heavy metals along with a 2–3 log reduction in air bacteria. In different study, Sun et al. [124] fabricated a chitosan-dipped nanostructured air filter and claim its ability to eliminate most bacteria from the air. Chitosan was used with bacterial cellulose to fabricate a nanostructured aerogel integrated with Ti-based metal–organic frameworks [125]. The bioaerogel had significantly high removal efficiencies of particulate matter even at low pressure drops. The authors stated that their fabrication had a filtration efficiency of more than 99.5% for PM2.5, with excellent stability even for a long time. Venkatesan et al. [126] evaluated a chitosan–alginate-based aerogel membrane as an air conditioner filter using an air-conditioner-like model and found that the chitosan–alginate filter showed a better performance than conventional commercial filters. The authors incorporated silver nanoparticles into their filtration system and reported a 1.5 times enhancement in filtration efficiency. Overall, chitosan nanostructured aerogels have great potential in particulate matter removal, and further research and development could lead to their widespread use in air filtration systems.

### 4.4. Alginate-Based Nanostructured Aerogel

**Figure 8:**
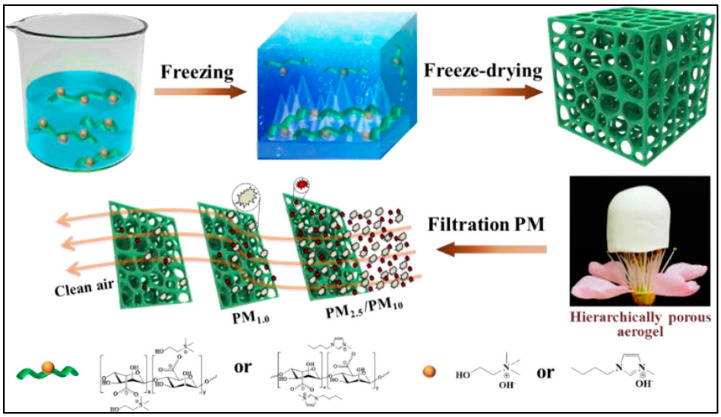
Schematic illustration of the fabrication of alginate-based aerogel for efficient removal of particulate matter. Adapted with permission from Wu et al. [131].

Alginate is a natural polysaccharide extracted from brown seaweed. It is a highly versatile biomaterial that has a wide range of applications in various fields, including air filtration and particulate matter removal [127]. Alginate-based aerogels has been studied as potential materials for air filtration due to their unique properties, including their high porosity, biodegradability, and ability to capture particles [128]. Alginate-based air filters work by using the electrostatic and adhesive properties of alginate to capture and trap airborne particles, such as fine dust, pollutants, and allergens. The alginate is often combined with other materials, such as activated carbon, to enhance its filtration efficiency and remove gases and odors. One of the advantages of alginate-based air filters is their low cost compared to other filter materials, making them a potentially affordable option for indoor air purification [129]. Additionally, alginate is a natural, biodegradable material that is considered safe for human use, which is a desirable property for air filtration applications. Deng et al. [130] fabricated a high-performance, ecofriendly, and biosafe PVA–sodium alginate–hydroxyapatite nanostructured composite using a green electrospinning technique. Owing to the nanopores in their fabrication, the authors were able to remove more than 99% of both types of fine particulate matter (PM0.3 and PM2.5). In order to improve its particulate matter capture efficiency, the authors created a unique wrinkled helical structure in their system, which promoted the physical interception of particulate pollutants. In a different study, Wu et al. [131] developed self-supporting nanostructured aerogels for efficient particulate matter removal. The authors introduced both organic or choline cations and 1-butyl-3-methylimidazolium into the alginate due to the electrostatic interaction, and finally, freeze drying in liquid N_2_ was used for the fabrication of the aerogel (Figure 8). The authors were able to remove up to 99.2%, 99.2%, and 93.4% of the PM10, PM2.5, and PM0.3 respectively. The aerogel performed this removal at low pressure drops of less than 10 Pa in a 15 h durability test. The use of alginate-based air filters is still in the early stages of research and development, and more studies are needed to fully understand their filtration efficiency and durability over time. Nonetheless, the potential of alginate in air filtration highlights the versatility of this biomaterial and its potential use in various applications.

## 5. Challenges of Nanostructured Bioaerogels in Particulate Matter Removal

Nanostructured bioaerogels have shown potential for use in air filtration and specifically particulate matter removal due to their high porosity; nanostructured pore size; and volume, low density, and biodegradability [132]. However, there are also some challenges associated with their use including limited mechanical strength, as most biopolymer aerogels including those derived from chitosan, cellulose, and alginate often exhibit low mechanical strength and can be easily damaged especially in humid conditions and where the filters are subject to mechanical stresses [133,134]. This can pose challenges during handling, transportation, and deployment in practical applications. The fragility of these materials may limit their use in environments with high airflows or turbulent conditions. The synthesis of nanostructured biopolymer aerogels is typically a complex and time-consuming process, involving sol–gel chemistry, supercritical drying, and post-treatment steps. Scaling up the production of these aerogels while maintaining their desirable properties can be challenging and expensive, hindering their widespread adoption for PM removal [135]. Although nanostructured biopolymer aerogels have a high surface area, their adsorption capacity for PM, especially for fine particles such as PM2.5 and nanoparticles, may be limited. Their adsorption performance depends on factors such as their specific surface chemistry, pore size distribution, and interparticle interactions, which need to be carefully optimized to enhance PM capture efficiency. Moisture absorption is another challenge of bioaerogels as most biopolymers have a high affinity for moisture, which can cause them to lose their structure and can reduce their filtration efficiency. This can be a particular challenge in high-humidity environments. Despite the great advances in the fabrication of bioaerogels, their manufacturing complexity is still a major challenge, especially in their large-scale production. The manufacturing process of biopolymer aerogels can be complex and may require specialized equipment and expertise [47]. To make biopolymer aerogels economically viable for PM removal, their reusability is crucial. However, regenerating these aerogels and restoring their original adsorption properties can be challenging. Techniques such as thermal, solvent, or chemical regeneration may be required, which can add complexity and cost to the overall process. The long-term stability of biopolymer aerogels is another concern. Exposure to moisture, UV radiation, and pollutants in the air can lead to degradation, structural collapse, or changes in surface chemistry, affecting their performance over time. Ensuring the stability and durability of these aerogels under real-world operating conditions is essential for their practical application [136]. The production of nanostructured biopolymer aerogels can involve expensive precursors, specialized equipment, and complex synthesis steps. These factors contribute to the overall cost of the materials, making them less economically viable for large-scale PM removal applications compared to other conventional filtration methods. Addressing these challenges requires further research and development efforts focused on improving the mechanical strength, scalability, adsorption capacity, regeneration methods, long-term stability, and cost effectiveness of nanostructured biopolymer aerogels for efficient particulate matter removal. The use of biopolymer aerogels in air filtration may offer a more sustainable and environmentally friendly alternative to traditional synthetic polymer-based filters. Biopolymer aerogels can be combined with other materials, such as activated carbon, to enhance their filtration efficiency.

## 6. Conclusions

Nanostructured biopolymer aerogels hold significant potential for PM removal due to their unique properties and versatile nature. These materials offer high porosity, large surface area, and tunable pore structure, which are crucial for efficient PM capture. Despite some challenges, ongoing research and development efforts are addressing these limitations and exploring ways to enhance their performance. The high porosity and large surface area of nanostructured biopolymer aerogels provide ample contact points for PM adsorption, allowing the effective removal of various particle sizes, including fine particles and nanoparticles. The composition and surface chemistry of these aerogels can be tailored to enhance their adsorption capacity for specific pollutants. Furthermore, the use of biopolymer aerogels derived from renewable sources such as chitosan, cellulose, and alginate offers an ecofriendly alternative to traditional filtration materials. The sustainability aspect of these materials aligns with the global drive toward green and environmentally friendly solutions for air pollution control. While there are challenges associated with their mechanical strength, scalability, regeneration, long-term stability, and cost, ongoing research is focusing on overcoming these limitations. Advances in material engineering, process optimization, and surface modification techniques are being explored to improve the mechanical properties and stability of biopolymer aerogels. Efforts are also underway to enhance their scalability and reduce production costs through innovative synthesis methods. In addition, the potential integration of nanostructured biopolymer aerogels with other filtration technologies or the development of composite materials can further enhance their PM removal efficiency. Synergistic effects between different materials can lead to improved performance, extending the application potential of biopolymer aerogels in PM control. Continued research, innovation, and collaboration between academia, industry, and policymakers will be crucial to unlock the full potential of nanostructured biopolymer aerogels and accelerate their practical implementation for cleaner and healthier air environments.

## Figures and Tables

**Table 1:** Illustration of nanostructured bioaerogel applications in different fields of study.

**Field**	**Application**	**Type of Aerogel**	**Remark**	**Ref.**
Medical	Drug delivery	Cellulose/sodium alginate aerogels	The aerogel showed sustained release of curcumin	[61]
	Tissue scaffolding	Nanocellulose/chitosan aerogel	The aerogel exhibited enhanced biocompatibility to human cells	[62]
	Wound dressing	Agar-based aerogel	The aerogel significantly shortened in vivo wound healing time	[63]
	Biosensing	Chitosan/carbon nanotube aerogel	The aerogel had multifunctional biosensing applications	[64]
Environmental	Air purification	Carbonized cellulose aerogel	The aerogel was able to remove all the PM2.5 and PM10	[65]
	Fertilizer delivery	Alginate-based aerogel	The aerogel exhibited sustained release of N-fertilizer	[66]
	Heavy metal removal	Chitosan-based aerogel	An effective adsorption and desorption of several heavy metals	[67]
	Water treatment	Green porous biochar aerogel	Complete removal of organic compounds was achieved from water	[68]
Industrial	Oil/water separation	Lignin-mediated fire-resistant aerogel	The aerogel was ultralight and had a high strength oil absorption property	[69]
	Protein separation	Nanofibrous aerogels	Super-elastic aerogel was prepared for efficient protein separation	[70]
	Food packaging	Nanocellulose/citrus pectin aerogel	The aerogels exhibited humidity control system for active packaging	[71]
	Thermal insulator	Cellulose nanofibril-based aerogel	The aerogel was highly flexible and had super thermal insulation properties	[72]
Others	Flame retardancy	Fully biomass-based aerogels	High flame retardancy was achieved in addition to excellent thermal insulation	[73]
	Supercapacitor	Cellulose carbon aerogel	High-performance supercapacitor was achieved	[74]
	Energy storage	Lignin/graphene/PEG aerogel	The aerogel showed efficient solar thermal energy storage	[75]

## Data Availability

Not applicable.
